# Views about responsibility for alcohol addiction and negative evaluations of naltrexone

**DOI:** 10.1186/s13011-015-0004-7

**Published:** 2015-03-08

**Authors:** Rebecca A Johnson, Jonathan M Lukens, Jonathan W Kole, Dominic A Sisti

**Affiliations:** Department of Sociology, Princeton University, 117 Wallace Hall, NJ 08544 Princeton, U.S.A; Salem State University, School of Social Work, 352 Lafayette Street, 01970 Salem, MA USA; Hasbro/Bradley’s Children’s Hospitals, Brown University, 593 Eddy St, Physician’s Office Building Suite 122, 02906 Providence, RI USA; Department of Medical Ethics & Health Policy, Perelman School of Medicine, University of Pennsylvania, 3401 Market Street, Suite 320, 19104 Philadelphia, PA USA

**Keywords:** Ethics, Alcohol addiction, Clinical practice patterns, Evidence-based medicine

## Abstract

**Background:**

Moral philosophers have debated the extent to which persons are individually responsible for the onset of and recovery from addiction. Empirical investigators have begun to explore counselors’ attitudes on these questions. Meanwhile, a separate literature has investigated counselors’ negative attitudes towards naltrexone, an important element of medication-assisted treatment for alcohol addiction. The present study bridges the literature on counselor views about responsibility for addiction with the literature on attitudes towards naltrexone. It investigates the extent to which a counselor’s views of individual responsibility for alcohol addiction are related to that counselor’s views of naltrexone.

**Methods:**

Using a vignette-based survey of 117 addiction treatment professionals, the study analyzes the relationship between an addiction counselor’s views about individual responsibility for alcohol addiction and using naltrexone to treat it.

**Results:**

We find a significant difference in counselors who assign greater responsibility to a person for the onset of alcohol addiction. They agreed more strongly with several objections to naltrexone, including worries about compliance, naltrexone’s side effects outweighing its benefits, naltrexone treating symptoms but not underlying causes, and the idea that medication may undermine a person’s motivation to recover. Combined views of greater responsibility for addiction’s onset and recovery also significantly predicted stronger agreement with objections.

**Conclusions:**

We conclude that there is a strong relationship between a counselor assigning higher individual responsibility for addiction and holding more negative views about naltrexone. The study also sheds light on one reason why the model of addiction as a brain disease has had limited impact on clinical practice.

## Background

Moral philosophers and addiction treatment professionals have explored the relationship between addiction and individual responsibility. Foddy and Savulescu [1] argue that conceptual models tend to characterize addiction in one of two ways: either as a symptom of a disease or as a failure of self-control. On the first account—the disease model of addiction—drug or alcohol use hijacks normal processes of motivation in the brain. Persons who continue to use drugs do so because biological changes limit their capacity to make rational choices. They may be individually responsible for their decision to initially use a substance, but after sufficient use, changes to their biological makeup make them no longer responsible for recovery. In contrast, the second account of addiction—the failure of self-control model—paints “addicts” as weak-willed persons who are locked in a battle for power over their pleasure-seeking impulses. In this model of addiction, persons are both individually responsible for their decision to use a substance and individually responsible for their recovery or failure to do so.

Recently, empirical researchers have begun to investigate the beliefs about responsibility that trained addiction counselors hold [2-5]. Studying U.S. addiction counselors, Kloss and Lisman [2] found that most counselors assigned low degrees of responsibility to persons for both the onset of and recovery from their alcohol addiction. Later studies of U.S. addiction counselors have investigated contributors to these attitudes. Counselors with higher rates of exposure to neuroscience and who identify as someone personally recovering from addiction hold a person significantly *less* responsible for addiction’s *onset* [4,5]*.* In contrast, counselors who identify cognitive behavioral therapy (CBT) as their theoretical orientation hold a person significantly *more* responsible for addiction’s onset, a finding the authors link to the responsibility-focused orientation of CBT [4]. Meanwhile, the only factor identified in the literature that significantly predicted assignments of responsibility for *recovery* from addiction is a counselor’s neuroscience exposure, with more exposure associated with assigning significantly more responsibility [4]. While these studies analyzed attitudes from U.S.-based addiction counselors, Palm’s study of a Swedish sample of addiction counselors found that counselors aligned with their U.S. counterparts in believing that persons are not highly responsible for the onset of their addiction. Yet the counselors diverged from their U.S. counterparts in believing that persons are highly responsible for recovery [3].

This literature has characterized counselor beliefs about responsibility for alcohol and other addictions, as well as characteristics of a counselor associated with those beliefs. Meanwhile, a separate literature has focused on the “implementation gap” in alcohol addiction treatment where centers fail to adhere to evidence-based treatment [6]. One way that centers deviate from evidence-based treatment is in their low uptake of naltrexone (Revia or Depade), a selective opioid antagonist that has been shown to be both safe and effective in decreasing the frequency and severity of relapse for persons with alcohol addiction [7,8]. Naltrexone in combination with psychosocial treatment is recommended as a first-line treatment for the condition [9]. Yet despite the scientific support for naltrexone, and despite its approval for alcohol addiction since 1995, prescriptions for naltrexone are low at both the level of treatment centers and individual counselors. The percentage of treatment facilities reporting *any* use of naltrexone has declined significantly since the drug was approved, declining from 49.2% in 1995 to 41.7% in 2004 [10]. Even among treatment programs that use naltrexone, only 3-9% of alcohol dependent clients received a prescription [10,11]. While it is true that naltrexone has small to medium effect sizes [12] and these are lower than some counselors would like [7], fewer than 1 in 10 clients receive a naltrexone prescription. Though not every client with alcohol addiction should receive a prescription, naltrexone still appears under-utilized relative to its effectiveness.

Research on reasons for this low utilization has identified several contributors. The primary reasons for a counselor’s reluctance to recommend naltrexone is inadequate knowledge of the medication and perceiving naltrexone to be high in cost [13]. Perceptions of adverse effects, worries that the local Alcoholics Anonymous chapter is opposed to medication, and a counselor’s geographic location (e.g. Tennessee) were also associated with greater reluctance to prescribe [13]. More recent research has confirmed that a counselor’s type of training plays a large role in support for naltrexone prescription—with psychiatrists and other physicians significantly more likely to support naltrexone and other medications than persons without medical degrees [14]. In the US, non MD counselors do not have the ability to prescribe and their clients may have limited access to a psychiatrist to prescribe naltrexone. Other recent research has illustrated that although counselors may *perceive* naltrexone as high in cost, this perception fails to align with the reality of naltrexone coverage, since most insurance plans make naltrexone affordable [15].

This research on low utilization of naltrexone has uncovered important contributors to low uptake. However, no published studies of which we are aware have bridged this literature on contributors to low naltrexone uptake with the literature on counselors’ views of individual responsibility for alcohol addiction. The present paper begins to bridge this gap. We focus on whether there is a relationship between a counselor’s views of individual responsibility for alcohol addiction and that counselor’s negative attitudes towards the use of naltrexone to treat the condition. While these negative attitudes are not the only factor influencing low uptake of medication, they may be one important barrier.

## Methods

### Recruitment methods

Participants were recruited through Qualtrics, a research software firm that provides access to panelists who agreed to be contacted to participate in relevant surveys. We used Qualtrics because in-person recruiting from a single addiction treatment facility or recruiting from a single geographic area (e.g. the New York metropolitan area) would likely result in a sample with a more narrow set of ideological views about addiction and clientele profile than recruiting from a national sample where the majority of states, insurance types, counselor educational backgrounds, and experience levels are represented. In addition, using a snowball sampling method, whereby we recruit personal contacts in the addiction treatment field and then these respondents refer others for recruitment, would likely also result in a significantly less diverse sample than those recruited from a national pool.

Qualtrics attempted to gear this survey towards panelists who indicated that they had experience treating persons with substance addictions. Following the University of Pennsylvania IRB decision that the study was exempt from full review, panelists were sent an email invitation to participate in a survey. Qualtrics sends out emails to persons who have registered to participate in survey research. The general Qualtrics pool includes 3.1 million people; for this specific survey, Qualtrics sent emails to members of that pool who, on their profiles, had indicated experience treating alcohol addiction. Because we wanted to ensure that our sample was limited to trained addiction counselors, our survey included further screening questions. The consent form specified that the survey was intended for a “*trained* addiction counselor who works with persons with various substance abuse addictions” (emphasis added). Participants were then asked to respond to the question “Do you currently or have you in the past treated persons with substance addictions?” and were screened out if they answered “no”. In line with payment levels for survey participants with particular professional experiences, respondents who completed the survey received $15 in payment. Participants completed the study in October 2012.

### Sample characteristics

Of the 382 potential participants who consented and responded to the screening question about whether they are treating or have treated persons with addiction, 255 persons (67%) responded “No” and were screened out of the survey while the remaining 127 persons (33%) answered “Yes” and were allowed to proceed. The final sample contained 117 participants who answered all the questions in the survey. Table 1 provides a summary of the sample. The mean age was 35 (SD = 11.32) and mean number of years working with persons with substance abuse was 2.5 (SD = 1.15). The insurance makeup of the clients treated by the counselor varied widely, but displayed a mean of 45.1% publicly insured clients (SD = 28.49), 42.6% privately insured clients (SD = 27.10), and a mean of 31.1% of clients paying out of pocket (SD = 30.21). Our sample’s composition of 48% BA/BS and MA-level counselors is acceptable for two reasons. First, PhD and MD-level professionals occupy a small minority of the addiction treatment workforce: fewer than 60% of addiction treatment programs even have a part-time physician on staff [16] and the highest degree for over 60% of persons who identify themselves as addiction counselors is a bachelor’s or master’s [17,18]. Second, since non-MD counselors are the most prevalent on addiction treatment staff, they can play multiple roles in medication-assisted treatment. First, counselors can refer clients to outside physicians with prescribing power. Second, although the primary reason that few centers have a physician on staff is inadequate insurance reimbursement for addiction, counselor attitudes towards the importance of medication might influence their willingness to advocate for physician hiring. Finally, we expected trained counselors to be aware of a medication that has been FDA approved for alcohol addiction since 1995 [19]. Therefore, we included counselors who are not physicians. A power analysis using G*Power suggested that for a regression with thirteen predictor variables, and that seeks a medium effect size difference in the dependent variable (Cohen’s f^2^ = 0.3), the study is 95% powered at 100 participants [20]. Therefore, the present study, with 117 participants, was adequately powered to detect differences in the variable of interest (strength of agreement with an objection to the use of naltrexone).

**Table 1:** **Demographic characteristics of survey respondents**

	**Total N = 117 (%)**
**Gender**	
Male	41 (35%)
Female	76 (65%)
**Age**	
18-30	43 (36.7%)
31-43	46 (39.3%)
44-56	20 (17.1%)
57+	8 (6.9%)
**Highest level of schooling**	
Ph.D. in clinical psychology	8 (7%)
Ed.D. (doctorate in education)	2 (2%)
M.D.	5 (4%)
Master of Social Work or Master of Education	16 (13%)
B.A. or B.S.	41 (35%)
High school or G.E.D.	31 (26%)
Other (included counseling certification, other Ph.D. or military substance abuse training)	14 (12%)

### Addiction vignette

First, participants read a vignette describing a client with alcohol addiction. The purpose of the vignette was to ensure that respondents had a relatively uniform “fictitious client” in mind when rating objections to the use of medication for that client.

The first paragraph of the vignette, which describes the symptoms of the person with alcohol addiction, was taken from Kloss and Lisman’s [2] study of addiction counselor attitudes towards the responsibility of a person with alcohol addiction depicted in a vignette. The original developers of the vignette constructed the symptoms based on the DSM-III-R depiction of psychoactive substance dependence (alcohol). In our adaptation of the vignette, we added three additional lines to describe the clinician’s choice of treatment (naltrexone and a 12-step program) and how another clinician at the treatment center raises objections to naltrexone’s use.

The vignette presents two counselors with conflicting viewpoints: one counselor who recommends naltrexone and a 12-step program for the client, Paul; another counselor who objects to the medication aspect of the treatment plan. Our reason for the first clinician recommending both naltrexone and a 12-step program is that most guidelines recommend combining naltrexone with some other form of counseling or support [21], and 12-step programs such as Alcoholics Anonymous have emphasized that they are not opposed to the use of medication [22]. We had three reasons for using the second clinician’s objections to the use of naltrexone as a starting point for investigating counselor attitudes. First was to contextualize the objections with reference to naltrexone’s use for the specific client in the vignette. Second was to present both positions on naltrexone (agreement with its use as in the first counselor; disagreement with its use as in the second counselor) as acceptable responses. Third was that asking participants about agreement with another counselor’s views minimized desirability biases if participants were aware that the study’s purpose was to examine the relationship between their view of responsibility for addiction and their objections to naltrexone.

In addition, the vignette was deliberately silent on certain details that might bias a counselor’s rating of the person’s degree of responsibility for the onset of/recovery from his addiction. It was also silent on details that could influence the counselor’s agreement with various objections to the use of naltrexone. For example, the vignette does not indicate how and why Paul started drinking. It does not analyze the extent to which he might be responsible for that decision. Finally, the vignette does not indicate whether or not Paul takes opioids for which naltrexone can cause withdrawal symptoms. In sum, the vignette is composed of a neutral presentation of symptoms that should not influence the respondents’ 1) views of addiction responsibility, or 2) attitudes towards naltrexone. Before fielding the vignette to respondents, we piloted it for face validity with persons trained in treating alcohol addiction:

*Paul goes to an addiction treatment center due to his inability to stop drinking without suffering withdrawal symptoms. He is having difficulty going to work and problems at home. He reports that at times, it is difficult for him to get out of bed in the morning. He reports having to drink twice as much as he used to just to feel "normal".*

*After a thorough evaluation, Paul is diagnosed with Psychoactive Substance Dependence (Alcohol). Paul’s clinician decides to treat him with Naltrexone (ReVia), a drug that claims to reduce or eliminate the rewarding effects of alcohol. He also recommends a 12-step program to Paul to attend. Another clinician at the clinic raises various objections to the use of Naltrexone for Paul’s treatment.*

### Attitudes toward the use of medication

After reading the vignette, participants were asked to rate the extent of their agreement on a 5-point Likert type scale (1 = strongly disagree to 5 = strongly agree) with six objections to the use of naltrexone to treat addiction raised by a “dissenting clinician” at the clinic. We focused on objections since the present study’s aim was to analyze views of responsibility for addiction that lead to more negative evaluations of naltrexone, which future work should supplement with attention to both negative and positive attitudes.

We surveyed the literature concerning counselor reluctance about naltrexone or other anti-addiction medications to develop the objections:

1. Paul will be unlikely to comply with taking the medication.
2. The medication’s side effects (which include nausea, headache, dizziness, anxiety and a small risk of withdrawal symptoms) are not worth its benefits.
3. There’s a risk that Paul will use opioids that interfere with the medication and cause serious side effects.
4. Naltrexone will treat the symptoms of Paul’s alcoholism, but it will not improve the underlying causes of his alcohol abuse.
5. Overcoming alcoholism without the use of medication will strengthen Paul’s resolve and willpower.
6. The medication will diminish Paul’s motivation to help himself and participate in the 12-step program.

Objection 1 stems from the fact that the most frequent physician-reported barrier to naltrexone prescription is worries about compliance [23]. Objection 2 originates in the finding that although physician focus groups do not perceive naltrexone’s side effects as a major barrier to its use, clients rank naltrexone’s side effect profile as one of the primary reasons why it is not more widely used [24]. Objection 3 is developed from the fact that naltrexone is contraindicated for clients currently dependent on opioids because of the severe withdrawal symptoms that may occur [25]. Objection 4 stems from views that trace the underlying causes of alcohol abuse to factors such as a lack of attractive opportunities available to persons with addiction or untreated psychiatric co-morbidities [26]; naltrexone, as a medication that targets pleasure derived from alcohol, does not address these perceived causes. Objection 5 stems from the fact that some counselors are reluctant to prescribe naltrexone to clients who seem unwilling to commit themselves to the “hard work” of treatment, a reluctance that appears rooted in the idea that a client can better strengthen his or her willpower through non-pharmacological treatment [23]. Objection 6 stems from a Family Physician Practice newsletter discussion of potential reasons behind reluctance to prescribe naltrexone. In the newsletter, a Harvard Medical School physician describes “a sense among many counselors that medications might diminish patients’ motivation to help themselves or to participate in 12-step program activities” [27]. These objections, drawn from existing research on negative attitudes towards naltrexone, vary in their degree of validity. For instance, rates of compliance for naltrexone range from 40% to 90%, making the objection somewhat valid depending on the rates with which the counselor is familiar. In contrast, most conclude that the side effects for naltrexone are generally mild and tolerable [12], making the objection largely invalid. What was important for the present study was not counselors’ *absolute degree* of agreement with each objection. Instead, the present study focuses on whether counselor views about responsibility significantly contributed to the *variation* in the counselors’ degree of agreement with each objection.

### Ratings of responsibility for the onset of and recovery from addiction

We assessed perceptions of a client’s responsibility for the onset of and recovery from addiction using a four-item scale adapted from Kloss and Lissman [2]. The items assessing views about responsibility for *onset* were:

1. Persons with alcoholism are responsible for the onset of their dependency.
2. Persons with alcoholism could have avoided their dependence.

The items assessing views about responsibility for *recovery* were:

1. Persons with alcoholism are personally responsible for their recovery.
2. Persons with alcoholism are personally responsible for creating a solution.

Respondents rated their extent of agreement on a 5-point Likert type scale (1 = strongly disagree to 5 = strongly agree). Responses to the two items about responsibility for onset displayed a significant bivariate correlation using a Pearson’s product–moment test (r = 0.48, n = 117, *p* < 0.001), so we combined the two items into a single responsibility for onset item that represented the average of each participant’s two responses. Responses to the items about responsibility for recovery also displayed a significant bivariate correlation using a Pearson’s product–moment test (r = 0.45, n = 117, *p* < 0.001), so the two items were combined and averaged into a single item. These items were used in model 2 and model 3 of our regressions respectively. In addition, a counselor’s combined view about addiction’s onset and his combined view about addiction’s recovery displayed a significant bivariate correlation using Pearson’s (r = 0.33, n = 117, p < 0.001). As a result, we created a combined “general views about responsibility” item that represented the average of these two responses and analyzed the item in model 4 of our regressions.

### Statistical analysis plan

We intended to treat each objection to naltrexone as a distinct dependent variable in our analyses. However, to test for any underlying factors for the six objections, we performed a principal components analysis using varimax rotation to see if any of the objections clustered together in a meaningful way. The analysis yielded 2 factors, but the factors only accounted for a combined total of 53.8% of the variance. Furthermore, five out of the six objections loaded onto one factor, with loading scores ranging from 0.43 (overcoming alcohol addiction will strengthen resolve) to 0.78 (the medication will diminish Paul’s motivation; the medication’s side effects are not worth its benefits). The second factor only included one item (medication will not improve the underlying cause of alcohol abuse). Therefore, we decided that the factors were not a useful means of clustering the objections to serve as dependent variables.

While the factor analysis revealed the objections did not meaningfully disaggregate into multiple scales, the Cronbach’s Alpha score for all of the objections was 0.63, with the objection about medication failing to treat addiction’s underlying cause the least similar to other objections. With that item removed, the scale’s Cronbach’s Alpha would have risen to 0.66. Since this still falls below the accepted Cronbach’s Alpha cutoff for scales of 0.70 [28], we decided to examine each objection to addiction medication as a *separate* dependent variable in separate regressions rather than combining them into a single scale. To account for the fact that we were performing multiple regressions (one set for each of the objections), we performed Bonferroni correction, resulting in a new cutoff of p < 0.008 for significance.

After examining distributional characteristics of the study factors, we used STATA version 13.1 (College Station, TX) to perform multiple regression analyses to examine the predictive effect of responsibility ratings on each objection to addiction medication. Each objection served as distinct continuous dependent variable that measured a counselor’s degree of agreement with the objection.

The predictor variables were the following demographic variables: gender (0 = female; 1 = male), age, type of schooling recoded into five distinct dummy variables (PhD; MD; Master’s; Bachelor’s; other education), with high school education-level as the reference category, years working with persons with substance abuse, percent of clients who are privately insured, percent of clients who are publicly insured, percent of clients who pay out of pocket, and personally recovering from addiction (0 = no; 1 = yes). For brevity, we call these the counselor-characteristics predictor variables. Model 1 examined these counselor-characteristic variables. Model 2 combined these counselor-characteristic variables with the counselor’s views about responsibility for the *onset* of addiction. Model 3 combined them with the counselor’s views about responsibility for *recovery* from the addiction. Model 4 combined these counselor-characteristic variables with a variable measuring a counselor’s combined view of responsibility for the *onset* of addiction and responsibility for *recovery* from addiction.

## Results

### Descriptive data on ratings of responsibility

Counselors rated the person depicted in the vignette as less responsible for the onset of his alcohol addiction (M = 3.33, SD = 0.95) than for recovery from his alcohol addiction (M = 3.81, SD = 0.84). A one-sample *t*-test showed that this difference in means was significant, *t*_*(116)*_ = 5.102, *p* < 0.001. Table 2 presents the breakdown of views of responsibility. The combined item measuring beliefs about responsibility for onset *and* beliefs about responsibility for recovery revealed that counselors assigned higher than neutral responsibility for addiction’s onset and higher than neutral responsibility for addiction’s recovery (M = 3.57, SD = 0.73).

**Table 2:** **Distribution of counselor views of responsibility and agreement with objections**

	**Strongly disagree**	**Disagree**	**Neither agree nor disagree**	**Agree**	**Strongly agree**
**Responsibility for onset**					
Persons with alcoholism are responsible for the onset of their dependency.	2.6%	24.8%	22.2%	35.0%	15.4%
Persons with alcoholism could have avoided their dependence.	5.1%	20.5%	29.1%	30.8%	14.5%
**Responsibility for recovery**					
Persons with alcoholism are personally responsible for their recovery.	1.7%	6.8%	6.0%	53.0%	32.5%
Persons with alcoholism are personally responsible for creating a solution.	3.4%	14.5%	23.9%	40.2%	17.9%
**Objection**					
Paul will be unlikely to comply with taking the medication.	1.7%	25.7%	35.9%	25.6%	11.1%
The medication’s side effects (which include nausea, headache, dizziness, anxiety and a small risk of withdrawal symptoms) are not worth its benefits.	8.5%	37.6%	19.7%	23.9%	10.3%
There’s a risk that Paul will use opioids that interfere with the medication and cause serious side effects.	12.0%	21.4%	22.2%	25.6%	18.8%
Naltrexone will treat the symptoms of Paul’s alcoholism, but it will not improve the underlying causes of his alcohol abuse.	2.5%	12.0%	26.5%	46.2%	12.8%
Overcoming alcoholism without the use of medication will strengthen Paul’s resolve and willpower.	1.7%	3.4%	18.0%	39.3%	37.6%
The medication will diminish Paul’s motivation to help himself and participate in the 12-step program.	6.8%	27.3%	19.7%	36.8%	9.4%

### Regression models

Tables 3, 4, 5, 6, 7 and 8 present the full results of the regression models for each of the six objections to the use of naltrexone for a person with alcohol addiction. In this section, we highlight the predictor variables that are the most consistent significant predictors of greater agreement with objections to medication. We also highlight *counselor-characteristic* variables that served as significant predictors of greater endorsement of objections.

**Table 3:** **Factors predicting objection that** ***persons will not comply with naltrexone***

	**Model 1**			**Model 2**			**Model 3**			**Model 4**		
**Predictor**	***B***	***SE B***	**β**	***B***	***SE B***	**β**	***B***	***SE B***	**β**	***B***	***SE B***	**β**
Gender (0 = female; 1 = male)	0.05	0.20	0.02	0.02	0.19	0.01	0.05	0.20	0.02	0.05	0.19	0.02
Age	0.02	0.01	0.18	0.02	0.01	0.19	0.02	0.01	0.18	0.02	0.01	0.18
Type of schooling												
PhD	−0.46	0.38	−0.13	−0.48	0.36	−0.13	−0.46	0.38	−0.13	−0.46	0.37	−0.13
MD	−0.19	0.48	−0.04	−0.39	0.47	−0.08	−0.19	0.49	−0.04	−0.17	0.47	−0.03
Master’s-level	−0.13	0.31	−0.04	−0.19	0.30	−0.07	−0.13	0.31	−0.04	−0.16	0.31	−0.06
Bachelor’s-level	−0.02	0.24	−0.01	−0.08	0.23	−0.04	−0.02	0.24	−0.01	−0.05	0.23	−0.03
Other education	0.11	0.34	0.03	0.15	0.33	0.05	0.11	0.34	0.03	0.14	0.34	0.05
Years working with substance abuse	−0.19*	0.09	−0.21*	−0.17	0.09	−0.19	−0.19	0.09	−0.21	−0.17	0.09	−0.20
Percentage privately insured clients	0.00	0.00	−0.03	0.00	0.00	−0.09	0.00	0.00	−0.03	0.00	0.00	−0.07
Percentage publicly insured clients	0.01	0.00	0.19	0.00	0.00	0.10	0.01	0.00	0.19	0.00	0.00	0.14
Percentage out of pocket clients	0.00	0.00	0.14	0.01	0.00	0.16	0.00	0.00	0.14	0.01	0.00	0.16
Personally recovering from addiction (0 = no; 1 = yes)	0.34	0.22	0.16	0.23	0.21	0.10	0.34	0.22	0.16	0.25	0.22	0.11
Responsibility for addiction *onset*				0.32**	0.10	0.31**						
Responsibility for addiction *recovery*							0.00	0.12	0.00			
Combined responsibility										0.27	0.14	0.20
*R* ^*2*^	0.16			0.23			0.16			0.19		
*F*	F (12, 104) = 1.68			F (13, 103) = 2.44**			F (13, 103) = 1.54			F (13, 103) = 1.89*		

*Notes:* for education, high school was reference group; *p ≤ 0.05, **p ≤ .01, ***p ≤ 0.001.

**Table 4:** **Factors predicting objection that** ***naltrexone’s side effects are not worth its benefits***

	**Model 1**			**Model 2**			**Model 3**			**Model 4**		
**Predictor**	***B***	***SE B***	**β**	***B***	***SE B***	**β**	***B***	***SE B***	**β**	***B***	***SE B***	**β**
Gender (0 = female; 1 = male)	0.14	0.24	0.06	0.11	0.23	0.04	0.16	0.24	0.07	0.13	0.23	0.06
Age	0.01	0.01	0.12	0.01	0.01	0.13	0.01	0.01	0.12	0.01	0.01	0.12
Type of schooling												
PhD	0.04	0.46	0.01	0.03	0.44	0.01	0.05	0.45	0.01	0.04	0.43	0.01
MD	0.06	0.59	0.01	−0.20	0.56	−0.04	0.30	0.58	0.05	0.09	0.55	0.02
Master’s-level	−0.25	0.38	−0.07	−0.33	0.36	−0.10	−0.27	0.37	−0.08	−0.33	0.36	−0.10
Bachelor’s-level	−0.12	0.29	−0.05	−0.19	0.28	−0.08	−0.13	0.28	−0.05	−0.18	0.27	−0.07
Other education	−0.17	0.42	−0.05	−0.11	0.40	−0.03	−0.13	0.41	−0.04	−0.09	0.40	−0.02
Years working with substance abuse	−0.15	0.11	−0.15	−0.13	0.11	−0.12	−0.14	0.11	−0.14	−0.13	0.11	−0.12
Percentage privately insured clients	0.00	0.00	0.06	0.00	0.00	−0.01	0.00	0.00	0.02	0.00	0.00	−0.02
Percentage publicly insured clients	0.00	0.00	0.11	0.00	0.00	0.01	0.00	0.00	0.09	0.00	0.00	0.02
Percentage out of pocket clients	0.01	0.00	0.17	0.01*	0.00	0.19*	0.01*	0.00	0.20*	0.01*	0.00	0.21*
Personally recovering from addiction (0 = no; 1 = yes)	0.30	0.26	0.12	0.15	0.26	0.06	0.19	0.26	0.07	0.09	0.26	0.04
Responsibility for addiction *onset*				0.42***	0.12	0.34***						
Responsibility for addiction *recovery*							0.33*	0.14	0.23*			
Combined Responsibility										0.59***	0.16	0.37***
*R* ^*2*^	0.09			0.18			0.14			0.20		
*F*	F (12, 104) = 0.85			F (13, 103) = 1.74			F (13, 103) = 1.25			F(13, 103) = 1.93*		

*Notes:* for education, high school was reference group; *p ≤ 0.05, **p ≤ .01, ***p ≤ 0.001.

**Table 5:** **Factors predicting objection that** ***persons will take opioids that interfere with naltrexone/are associated with serious side effects***

	**Model 1**			**Model 2**			**Model 3**			**Model 4**		
**Predictor**	***B***	***SE B***	**β**	***B***	***SE B***	**β**	***B***	***SE B***	**β**	***B***	***SE B***	**β**
Gender (0 = female; 1 = male)	0.12	0.26	0.04	0.10	0.26	0.04	0.14	0.26	0.05	0.12	0.26	0.04
Age	−0.01	0.01	−0.07	−0.01	0.01	−0.06	−0.01	0.01	−0.07	−0.01	0.01	−0.07
Type of schooling												
PhD	0.76	0.50	0.16	0.75	0.50	0.16	0.76	0.50	0.17	0.76	0.49	0.16
MD	1.11	0.64	0.17	0.94	0.64	0.15	1.28	0.65	0.20	1.13	0.63	0.18
Master’s-level	0.67	0.42	0.18	0.62	0.41	0.16	0.66	0.41	0.17	0.62	0.41	0.16
Bachelor’s-level	0.45	0.32	0.17	0.40	0.31	0.15	0.44	0.31	0.16	0.41	0.31	0.15
Other education	0.54	0.46	0.14	0.58	0.45	0.15	0.57	0.45	0.14	0.60	0.45	0.15
Years working with substance abuse	0.00	0.12	0.00	0.01	0.12	0.01	0.00	0.12	0.00	0.01	0.12	0.01
Percentage privately insured clients	0.00	0.00	0.04	0.00	0.00	0.00	0.00	0.00	0.02	0.00	0.00	−0.01
Percentage publicly insured clients	0.01	0.00	0.12	0.00	0.00	0.06	0.00	0.00	0.10	0.00	0.00	0.06
Percentage out of pocket clients	0.01*	0.00	0.20*	0.01*	0.00	0.21*	0.01*	0.00	0.22*	0.01*	0.00	0.23*
Personally recovering from addiction (0 = no; 1 = yes)	0.09	0.29	0.03	0.00	0.29	0.00	0.02	0.29	0.01	−0.04	0.29	−0.02
Responsibility for addiction *onset*				0.28	0.14	0.20						
Responsibility for addiction *recovery*							0.23	0.15	0.15			
Combined responsibility										0.40*	0.18	0.22*
*R* ^*2*^	0.12			0.17			0.14			0.16		
*F*	F (12, 104) = 1.17			F(13, 103) = 1.40			F (13, 103) = 1.27			F(13, 103) = 1.49		

*Notes:* for education, high school was reference group; *p ≤ 0.05, **p ≤ .01, ***p ≤ 0.001.

**Table 6:** **Factors predicting objection that** ***naltrexone will treat the symptoms of alcohol abuse, but not its underlying causes***

	**Model 1**			**Model 2**			**Model 3**			**Model 4**		
**Predictor**	***B***	***SE B***	**β**	***B***	***SE B***	**β**	***B***	***SE B***	**Β**	***B***	***SE B***	**β**
Gender (0 = female; 1 = male)	−0.38*	0.18	−0.19*	−0.40*	0.18	−0.20*	−0.37*	0.18	−0.19*	−0.38*	0.18	−0.19*
Age	−0.01	0.01	−0.11	−0.01	0.01	−0.10	−0.01	0.01	−0.11	−0.01	0.01	−0.10
Type of schooling												
PhD	0.66	0.35	0.19	0.65	0.34	0.19	0.66	0.35	0.20	0.66	0.34	0.19
MD	0.37	0.45	0.08	0.20	0.44	0.04	0.52	0.45	0.11	0.39	0.43	0.08
Master’s-level	0.33	0.29	0.12	0.27	0.28	0.10	0.31	0.29	0.11	0.28	0.28	0.10
Bachelor’s-level	0.28	0.22	0.14	0.23	0.22	0.12	0.27	0.22	0.13	0.24	0.21	0.12
Other education	0.59	0.32	0.20	0.63*	0.31	0.22*	0.62	0.32	0.21	0.65*	0.31	0.22*
Years working with substance abuse	0.02	0.09	0.02	0.03	0.08	0.04	0.02	0.09	0.02	0.03	0.08	0.04
Percentage privately insured clients	0.01*	0.00	0.25*	0.01*	0.00	0.19*	0.01	0.00	0.22	0.01	0.00	0.18
Percentage publicly insured clients	0.00	0.00	−0.12	−0.01*	0.00	−0.20*	0.00	0.00	−0.14	−0.01*	0.00	−0.20*
Percentage out of pocket clients	0.01*	0.00	0.25*	0.01**	0.00	0.26**	0.01**	0.00	0.27**	0.01**	0.00	0.28**
Personally recovering from addiction (0 = no; 1 = yes)	−0.05	0.20	−0.02	−0.14	0.20	−0.07	−0.12	0.20	−0.06	−0.18	0.20	−0.08
Responsibility for addiction *onset*				0.27**	0.10	0.27**						
Responsibility for addiction *recovery*							0.21*	0.11	0.19*			
Combined responsibility										0.38**	0.13	0.29**
*R* ^*2*^	0.19			0.25			0.22			0.26		
*F*	F (12, 104) = 2.05*			F(13, 103) = 2.59**			F(13, 103) = 2.25*			F(13, 103) = 2.74**		

*Notes:* for education, high school was reference group; *p ≤ 0.05 ** *p* ≤ .01, *** *p* ≤ 0.001.

**Table 7:** **Factors predicting objection that** ***overcoming alcoholism without medication strengthens a person’s resolve/willpower***

	**Model 1**			**Model 2**			**Model 3**			**Model 4**		
**Predictor**	***B***	***SE B***	**β**	***B***	***SE B***	**β**	***B***	***SE B***	**β**	***B***	***SE B***	**β**
Gender (0 = female; 1 = male)	−0.26	0.19	−0.13	−0.26	0.19	−0.14	−0.24	0.19	−0.12	−0.26	0.19	−0.13
Age	0.01	0.01	0.11	0.01	0.01	0.12	0.01	0.01	0.11	0.01	0.01	0.12
Type of schooling												
PhD	0.12	0.37	0.04	0.12	0.37	0.04	0.13	0.36	0.04	0.12	0.36	0.04
MD	−0.32	0.47	−0.07	−0.35	0.47	−0.08	−0.09	0.46	−0.02	−0.30	0.46	−0.07
Master’s-level	0.00	0.30	0.00	−0.01	0.31	−0.01	−0.02	0.29	−0.01	−0.04	0.30	−0.01
Bachelor’s-level	0.10	0.23	0.05	0.09	0.23	0.05	0.09	0.22	0.05	0.07	0.23	0.04
Other education	−0.01	0.33	−0.01	−0.01	0.33	0.00	0.02	0.32	0.01	0.02	0.33	0.01
Years working with substance abuse	−0.01	0.09	−0.02	−0.01	0.09	−0.01	−0.01	0.09	−0.01	0.00	0.09	0.00
Percentage privately insured clients	0.00	0.00	−0.03	0.00	0.00	−0.04	0.00	0.00	−0.07	0.00	0.00	−0.07
Percentage publicly insured clients	0.00	0.00	0.11	0.00	0.00	0.09	0.00	0.00	0.08	0.00	0.00	0.05
Percentage out of pocket clients	0.00	0.00	−0.04	0.00	0.00	−0.03	0.00	0.00	0.00	0.00	0.00	−0.01
Personally recovering from addiction (0 = no; 1 = yes)	−0.13	0.21	−0.07	−0.15	0.21	−0.07	−0.23	0.21	−0.12	−0.22	0.21	−0.11
Responsibility for addiction *onset*				0.05	0.10	0.05						
Responsibility for addiction *recovery*							0.30**	0.11	0.28**			
Combined responsibility										0.26	0.13	0.21
*R* ^*2*^	0.06			0.07			0.13			0.10		
*F*	F(12, 104) = 0.62			F(13, 103) = 0.58			F(13, 103) = 1.21			F(13, 103) = 0.88		

*Notes:* for education, high school was reference group; *p ≤ 0.05, **p ≤ .01, ***p ≤ 0.001.

**Table 8:** **Factors predicting objection that** ***the medication will diminish a person’s motivation to help himself/participate in the 12-step program***

	**Model 1**			**Model 2**			**Model 3**			**Model 4**		
**Predictor**	***B***	***SE B***	**β**	***B***	***SE B***	**β**	***B***	***SE B***	**β**	***B***	***SE B***	**β**
Gender (0 = female; 1 = male)	−0.10	0.24	−0.04	−0.13	0.23	−0.05	−0.09	0.24	−0.04	−0.10	0.23	−0.04
Age	−0.01	0.01	−0.06	−0.01	0.01	−0.05	−0.01	0.01	−0.06	−0.01	0.01	−0.06
Type of schooling												
PhD	0.22	0.45	0.05	0.21	0.44	0.05	0.22	0.45	0.06	0.22	0.44	0.05
MD	−0.02	0.57	0.00	−0.24	0.56	−0.04	0.06	0.58	0.01	0.00	0.56	0.00
Master’s-level	−0.29	0.37	−0.09	−0.36	0.36	−0.11	−0.30	0.37	−0.09	−0.34	0.37	−0.10
Bachelor’s-level	−0.13	0.28	−0.05	−0.19	0.28	−0.08	−0.13	0.28	−0.06	−0.17	0.28	−0.07
Other education	−0.24	0.41	−0.07	−0.19	0.40	−0.05	−0.22	0.41	−0.06	−0.19	0.40	−0.05
Years working with substance abuse	−0.02	0.11	−0.02	0.00	0.11	0.00	−0.01	0.11	−0.01	0.00	0.11	0.00
Percentage privately insured clients	0.01	0.00	0.16	0.00	0.00	0.10	0.01	0.00	0.15	0.00	0.00	0.11
Percentage publicly insured clients	0.00	0.00	0.04	0.00	0.00	−0.05	0.00	0.00	0.03	0.00	0.00	−0.03
Percentage out of pocket clients	0.00	0.00	0.10	0.00	0.00	0.12	0.00	0.00	0.11	0.00	0.00	0.13
Personally recovering from addiction (0 = no; 1 = yes)	−0.07	0.26	−0.02	−0.19	0.25	−0.08	−0.11	0.26	−0.04	−0.20	0.26	−0.08
Responsibility for addiction *onset*				0.35**	0.12	0.29**						
Responsibility for addiction *recovery*							0.11	0.14	0.08			
Combined responsibility										0.37*	0.16	0.24*
*R* ^*2*^	0.07			0.13			0.07			0.11		
*F*	F (12, 104) = 0.62			F(13, 103) = 1.21			F(13, 103) = 0.62			F(13, 103) = 1.00		

*Notes:* for education, high school was reference group; *p ≤ 0.05, **p ≤ .01, ***p ≤ 0.001.

The counselor-characteristic variables (Model 1 in Tables 3, 4, 5, 6, 7 and 8) largely failed to significantly predict greater or lesser agreement with the objections to naltrexone. In addition, the *R*^*2*^ values reported in Tables 3, 4, 5, 6, 7 and 8 for Model 1 highlight that the variables failed to explain a large proportion of the variance in this agreement. The only counselor characteristic that was a significant predictor of greater agreement with an objection when views about responsibility were added in Models 2–4 was the counselor treating a higher percentage of out of pocket clients. This predicted significantly greater agreement with the objection that naltrexone will treat the symptoms of alcohol abuse but not its underlying causes (Table 6), when examined in combination with views of responsibility for *onset* (β = 0.26, t(103) = 2.94, p = 0.004), for *recovery* (β = 0.27, t(103) = 2.94, p = 0.004), and with combined views (β = 0.28, t(103) = 3.11, p = 0.002). This characteristic also predicted stronger agreement with two other objections (medication’s side effects are not worth its benefits, and the person will take opioids that interfere), but at the p < 0.05 level rather than our Bonferroni adjusted cutoff of p < 0.008 (Table 4; Table 5).

In contrast to counselor characteristics that largely failed to predict strength of agreement with objections, a counselors subjective views about responsibility showed predictive relationships that were both more significant and more consistent across objections to the use of naltrexone for alcohol addiction. More specifically, a counselor assigning *higher* responsibility to a person for the *onset* of their addiction (Model 2) significantly predicted stronger agreement with 1) the idea that persons are unlikely to comply with naltrexone (β = 0.31, t(103) = 3.13, p = 0.002) (Table 3), 2) naltrexone’s side effects are not worth its benefits (B = 0.34, t(103) = 3.39, p = 0.001). (Table 4), 3) naltrexone treats alcohol abuse’s symptoms but not its underlying cause (β = 0.27, t(103) = 2.75, p = 0.007) (Table 6), and 4) medication will diminish a person’s motivation to help themselves (β = 0.29, t(103) = 2.80, p = 0.006) (Table 8). Views about a person’s responsibility for *recovery* from addiction (Model 3) were less predictive, only significantly predicting stronger agreement with the idea that overcoming alcoholism without medication strengthens resolve and willpower (β = 0.28, t(103) = 2.79, p = 0.006) (Table 7). The combined measure of a person’s responsibility for *onset* and for *recovery* predicted significantly stronger agreement with two objections: 1) naltrexone’s side effects are not worth its benefits (β = 0.37, t(103) = 3.69, p < 0.001) (Table 4) and 2) naltrexone treats alcohol abuse’s symptoms but not its underlying cause (β = 0.29, t(103) = 3.02, p = 0.003) (Table 6). However, this effect seems to be driven largely by views of responsibility for onset rather than views of responsibility for recovery.

## Discussion

Results from our vignette-based study of addiction counselors indicate that a counselor’s assignment of greater responsibility for alcohol addiction is associated with stronger agreement with various objections to the use of a medication—naltrexone—for alcohol addiction treatment. In particular, counselors who assign a person greater degrees of responsibility for addiction’s onset display stronger agreement to four of the six objections to naltrexone we investigated. These objections included both issues related to naltrexone’s efficacy, such as the medication’s side effects not being worth its benefits, as well as concerns that medication might diminish a person’s motivation to help themselves. While holding a person more responsible for the *onset* of addiction was associated with several objections, beliefs about a person’s responsibility for *recovery* were less predictive. The study, one of the first to link the literature on counselor beliefs about responsibility for addiction with the literature on counselor attitudes towards naltrexone, thus illustrates the presence of a link between these two types of counselor attitudes.

In analyzing these findings, we focus on three sets of questions. First, what do the particular objections to the use of medication that counselors endorse reveal about the relationship between responsibility-focused views of addiction and negative evaluations of naltrexone? Second, why does the counselor’s percentage of out-of-pocket clients also matter in their skepticism towards medication? Third, how do the findings relate to the broader literature on the limited uptake of the “brain disease” model of addiction among clinical practitioners?

### Views of responsibility and clinical evidence

Interestingly, many of the objections that counselors more strongly endorse could be characterized as clinical in nature rather than deep-seated opposition to the idea of using *any* medication to treat addiction. These include worries about compliance and side effects. Each of these objections has some empirical support in the clinical literature on naltrexone, but presumably, counselors with different views of responsibility should assess this clinical evidence in similar ways. The fact that counselors in our sample with different views of responsibility for addiction assessed the evidence in significantly different ways presents an interesting finding about the interaction between attributions of addiction responsibility and the weighing of evidence.

The present study only examined *that* counselors’ negative attitudes towards naltrexone are linked to holding persons more individually responsible for addiction’s onset. Future research should investigate mechanisms *by which* these views become linked. One possible mechanism that future research should investigate include: responsibility-oriented treatment professionals may attune themselves more to articles they read on the unpleasant side effects of a medication, data on lack of compliance among clients, and information on negative drug-drug interactions, than counselors who are less responsibility-oriented and more inclined towards positive evaluations of medication.

### Client insurance status and skepticism towards medication

Second, the present study found no significant predictive roles for certain counselor characteristics that other research finds either contribute to views of responsibility or contribute to reluctance to prescribe naltrexone: for example, that counselors who are personally recovering from addiction hold persons less responsible for the condition [4] or that counselors who treat a larger percentage of Medicaid or uninsured clients display lower support for medication [23]. Instead, we found that a counselor treating a higher percentage of clients who pay out of pocket for treatment significantly predicted greater agreement with the objection that the medication may treat the symptoms of alcohol abuse but not its underlying cause. What might be the link between treating more out-of-pocket clients and exhibiting greater skepticism towards medication?

Clients may pay out of pocket for addiction treatment for several reasons: they may lack insurance or have insurance but do not want addiction treatment to appear in their billing records. However, clients may also pay out of pocket if they seek care at one of the estimated 50-60% of alcohol and substance addiction centers that do not accept any form of insurance [29]. Indeed, past research suggests that treatment programs may be split between ones that adopt an abstinence-only philosophy that eschews medication and ones that promote medication-assisted treatment [30]. Though further research is needed, it is possible that these organizations that deliberately do not accept insurance have chosen to opt for a highly responsibility-focused view of addiction that rejects both medical insurance and medication. Future work should explore this possibility by collecting data on the reimbursement policies of the treatment centers where counselors work.

### The gap between research and practice

Third, this study adds to a growing body of evidence showing that the “chronic relapsing brain disease” model of addiction, though promoted by research leaders in the addiction community [31], has failed to fully penetrate clinical practice. This is in spite of support for medication-assisted treatment among many addiction scientists and clients [32].

The present study showed that counselors who hold responsibility-focused views of addiction view one achievement of the brain disease model of addiction—naltrexone—more negatively than counselors with less responsibility-focused views. Past qualitative research has shown that some practicing counselors report avoiding conveying the brain disease model of addiction to clients out of fear that clients will fail to take “personal responsibility for what’s happening to them” [33]. Supplementing these interview-based studies are accounts from within addiction treatment centers that illustrate the centers’ focus on instilling ideas of responsibility into clients and de-emphasis on medications like naltrexone as a part of treatment [34]. The present study complements these qualitative accounts by providing quantitative evidence of a link between two sets of attitudes that might inhibit uptake of evidence-based medication.

### Limitations

The current study has several limitations. First, although there were significant correlations between provider’s views about responsibility and attitudes towards addiction, our design neither isolated causality nor confirmed directionality. We did not inquire about negative and positive past experiences with naltrexone, but future research should more closely analyze whether views of greater responsibility for addiction tend to precede and possibly cause more negative evaluations of naltrexone, or whether more negative clinical experiences with naltrexone may result in believing that persons ought to be more responsible for addiction. In addition, these studies should include both objections *to* naltrexone and reasons *for* the use of naltrexone. Second, the use of only a single case limited our understanding of subtleties in counselor attitudes towards naltrexone. There may be some clients or scenarios where providers have greater or lesser confidence in pharmacologic assistance in overcoming addiction. This study examined general provider attitudes towards naltrexone, but more detailed instruments could better illuminate nuances in counselor views.

Third, we did not assess the organizational context in which our respondents worked, which past research has shown can influence views about medication [35] and which may also influence views about responsibility for addiction. For example, respondents from a hospital system, working with medically oriented colleagues, may have endorsed fewer concerns with naltrexone simply due to greater familiarity with the medication. In contrast, counselors in 12-step oriented institutions may have minimal experience with naltrexone and thus are uncomfortable with potential side effects and interactions. More research on the effects of organizational context on both understanding of naltrexone and views of addiction responsibility is needed. Fourth, while there was some diversity of training in our cohort, it was largely composed of persons with bachelor’s and master’s degrees working at addiction treatment facilities as counselors. This stemmed from our choice to recruit a sample that reflected the variety of backgrounds in the addiction treatment workforce. In addition, we did not measure the caseload mix that the counselor treats (e.g. only alcohol addiction; a mix of alcohol addiction and drug dependence), which may influence the counselor’s knowledge about and attitudes towards naltrexone. Future research could explore whether the findings hold true for physician-only samples and for counselors with a variety of caseloads.

Fifth, our desire for a diverse participant pool led to the use of an online survey. Online surveys run a small risk that persons might misrepresent their professional credentials. However, the consent form reiterated that the study was only intended for trained addiction treatment professionals, hopefully minimizing this possibility. Sixth, although Qualtrics Panels recruits from a large sample of potential respondents, there may be biases in who opts in to the panel in general and then to specific surveys. Future research should confirm our findings with efforts to recruit a random, representative sample of the U.S. addiction treatment workforce.

## Conclusion

The present study finds a link between a counselor assigning a higher degree of individual responsibility to a person for his addiction and greater endorsement of several objections to the use of naltrexone. Future research should investigate factors such as which negative evaluations most strongly predict a reluctance to *prescribe* naltrexone, how different addiction treatment organizations socialize counselors to adopt certain attitudes about responsibility for addiction, and how positive and negative experiences with naltrexone shape these views. Future research should also extend these investigations to the injectable form of naltrexone and other anti-addiction medications. These studies can help inform interventions that address low uptake of medication-assisted treatment among the addiction treatment workforce.
